# Reduction and Oxidation of Cu Species in Cu-Faujasites Studied by IR Spectroscopy

**DOI:** 10.3390/molecules25204765

**Published:** 2020-10-16

**Authors:** Łukasz Kuterasiński, Jerzy Podobiński, Ewa Madej, Małgorzata Smoliło-Utrata, Dorota Rutkowska-Zbik, Jerzy Datka

**Affiliations:** Jerzy Haber Institute of Catalysis and Surface Chemistry, Polish Academy of Sciences, Niezapominajek 8, 30-239 Krakow, Poland; nckutera@cyf-kr.edu.pl (Ł.K.); ncpodobi@cyf-kr.edu.pl (J.P.); nczackie@cyf-kr.edu.pl (E.M.); malgorzata.smolilo-utrata@ikifp.edu.pl (M.S.-U.); nczbik@cyf-kr.edu.pl (D.R.-Z.)

**Keywords:** CO/NO-IR spectroscopy, Cu sites, reduction, hydrogen, ethanol

## Abstract

The process of reduction (by hydrogen and ethanol) and oxidation (by oxygen and NO) of Cu sites in dealuminated faujasite-type zeolites (of Si/Al = 31) was studied by infrared (IR) spectroscopy with CO (for Cu^+^) and NO (for Cu^2+^) as probe molecules. Two zeolites were studied: one of them contained mostly Cu^+^_exch._, whereas another one contained mostly Cu^2+^ and Cu^+^_ox._ The susceptibility of various forms of Cu for reduction were investigated. IR experiments of CO sorption evidenced that Cu^+^_ox._ was more prone for the reduction than Cu^+^_exch._ According to NO sorption studies, Cu^2+^_exch._ was reduced in the first order before Cu^2+^_ox._ Ethanol reduced mostly Cu^2+^ and, also, some amounts of Cu^+^. The treatment with oxygen caused the oxidation of Cu^+^ (both Cu^+^_exch._ and Cu^+^_ox._) to Cu^2+^. The adsorption of NO at 190K produced Cu^+^(NO)_2_ dinitrosyls, but heating to room temperature transformed dinitrosyls to mononitrosyls and increased the Cu^2+^ content.

## 1. Introduction

Copper-exchanged zeolites are used as active and selective catalysts for many chemical reactions, such as: NO_x_ abatement, oxidation, isomerization, dehydration and many other processes [1,2,3,4,5,6,7,8,9,10,11]. Attractive catalytic properties of these materials can be related to both the various oxidation states and the occurrence of copper species in different forms [12]. Our present study concerns Cu species in zeolites Y. Based on the analysis of results from many experimental techniques, it was found that the Cu^2+^ ions were located in the S_I_ sites (inside hexagonal prisms) and were characterized by the lowest tendency to reduction among the all existing copper species in this type of zeolitic structure [13]. The status of copper species was often determined by infrared (IR) studies of CO adsorption. The choice of CO as a probe molecule for Cu ions is implied from the existence of a characteristic carbonyl frequency shift, which depends strictly on the copper coordination environment, as well as the oxidation state [14,15,16]. In the case of Cu^2+^ cations, the CO-IR analysis is insufficient, thus complementary techniques such as Electron Paramagnetic Resonance (EPR) [17,18,19], X-ray photoelectron spectroscopy (XPS) [20,21,22] and temperature-programmed reduction (TPR) [20,23,24,25,26], as well as IR studies of NO sorption, are needed to receive a detailed characterization of the oxidation state and properties of Cu in zeolites under various red-ox conditions, e.g., [27,28,29].

Campos-Martin et al. [20] studied the location of Cu in copper-loaded Y-type zeolites by TPR, CO-Fourier-transform infrared (FTIR) and XPS methods. Samples outgassed at 673 K contained Cu^+^ produced by the reduction of Cu^2+^ in vacuum. In the case of CuY zeolite, for which Cu was introduced by the ionic-exchange method, copper was found in the exchange positions, while in the impregnated sample, Cu^2+^ and Cu^+^ ions were located mostly on the surface of CuO crystals, and small proportions of Cu^+^ in accessible exchange sites S_II_ and S_II*_ were found. Sites S_II_ and S_II*_ are situated inside supercages out of plane and in plane of the hexagonal oxygen ring, respectively. Samples reduced in hydrogen at 523 K contained Cu^0^ species in the impregnated samples, while Cu^+^ prevailed in the exchanged analogs. Reduction at 598 K caused the reduction of a significant part of the Cu^+^ species to Cu^0^, with a simultaneous migration of Cu^+^ to S_II*_ sites. It was also shown that Cu^2+^ or Cu^+^ were present in outgassed samples, while for the samples reduced in H_2_ at 623 K, only Cu^0^ and intrazeolite Cu^+^ were found.

The present study is the continuation of our earlier investigation [27] in which the status and properties of Cu ions in zeolites of the faujasite (FAU) type of Si/Al = 31 were followed by IR spectroscopy, with CO and NO as the probe molecules. Cu was introduced [27] by the impregnation method to zeolites in the protonic (HFAU) or sodium form (NaFAU). Cu was in the form of Cu^+^ in the exchange form (Cu^+^_exch._), Cu^+^ in the oxide form (Cu^+^_ox._) and as Cu^2+^ (mostly CuO). The proportion between the amounts of these Cu forms depended on the amount of Cu in zeolites and the form of zeolite to which Cu was introduced: HFAU or NaFAU. Zeolites CuHFAU contained mostly Cu^+^_exch._ and only small amounts of Cu^+^_ox._ and Cu^2+^_._ On the other hand, zeolites CuNaFAU contained much smaller amounts of Cu^+^_exch._ and much bigger contributions of Cu^+^_ox._ and Cu^2+^. Both Cu^+^ and Cu^2+^ in oxide forms showed stronger electron donor properties than Cu^+^ and Cu^2+^ in the exchange positions.

As mentioned above, in the present study, we followed the reduction by hydrogen and by ethanol, as well as oxidation by the oxygen of Cu sites in Cu-faujasites. IR spectroscopy with CO and NO as the probe molecules was our main experimental method, but X-ray absorption spectroscopy (XAS) and X-ray diffraction (XRD) were also applied as the supplementary methods.

## 2. Results and Discussion

### 2.1. Cu Species in Cu(2)HFAU and Cu(5)NaFAU

**Figure 1:**
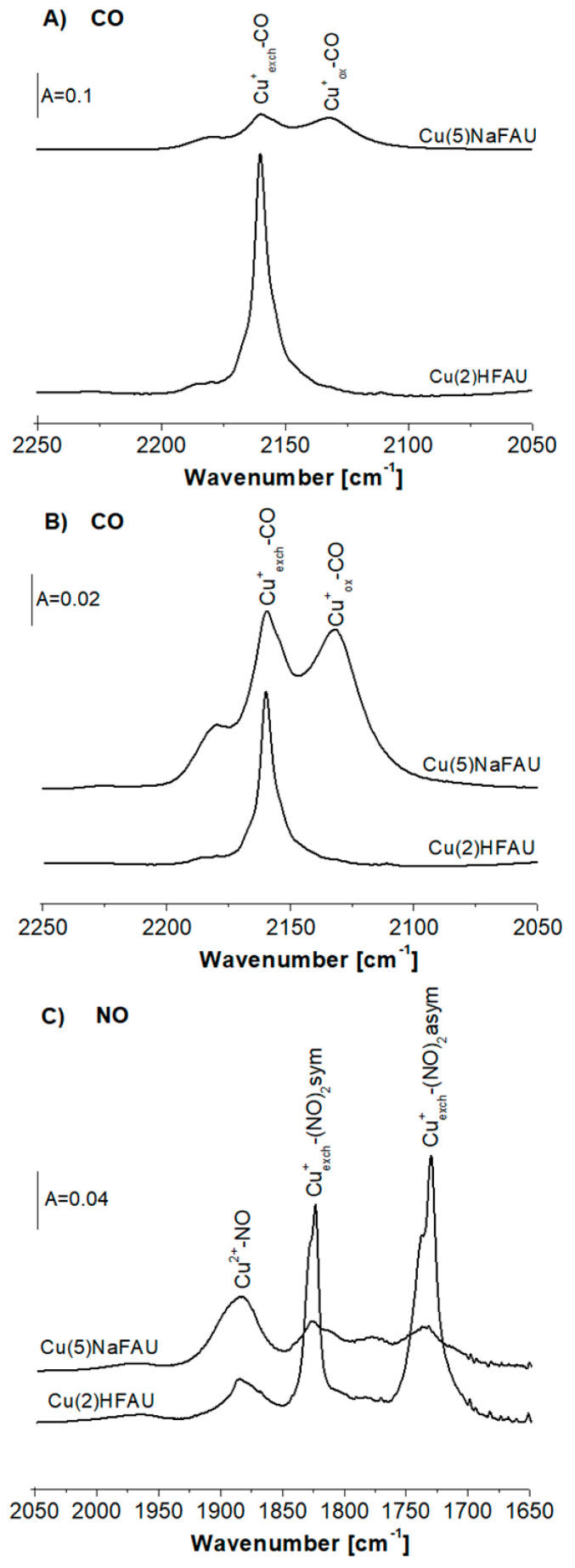
(**A**,**B**) The infrared (IR) spectra of CO sorbed at room temperature in Cu(2)HFAU and Cu(5)NaFAU zeolites. Spectra are normalized to 10 mg of the sample (**A**) and to the band intensity (**B**). (**C**) The IR spectra of NO sorbed at 190 K on Cu(2)HFAU and Cu(5)NaFAU zeolites.

The IR spectra of CO sorbed at room temperature in Cu(2)HFAU and Cu(5)NaFAU zeolites are presented in Figure 1A. The CO bands are significantly smaller for Cu(5)NaFAU, indicating that the amounts of Cu^+^ accessible to CO molecules is also lower, despite a higher Cu content (5 wt.% vs. 2 wt.% in Cu(2)HFAU). This difference was explained in our previous paper [27] as the result of a different procedure of introduction of Cu to both zeolites. Zeolite Cu(2)HFAU was obtained by the impregnation of HFAU (Si/Al = 31) with Cu(NO_3_)_2_ that caused the production of CuHFAU and HNO_3_, which was removed during calcination, which was done upon the impregnation procedure. It shifted the exchange equilibrium right towards the formation of CuHFAU. On the other hand, the impregnation of NaFAU with Cu(NO_3_)_2_ produces NaNO_3_, which is not removed by calcination, and it shifts the equilibrium back (towards NaFAU). Therefore, the amount of Cu introduced into exchange positions is small in this zeolite. Most of Cu (Cu^+^ and Cu^2+^) is in oxide forms. Probably such oxides are in cluster forms, with only a small fraction of Cu being on the surface and accessible to probe molecules The spectra of CO interacting with Cu^+^ sites in Cu(2)HFAU and Cu(5)NaFAU zeolites normalized to the same band intensity are presented in Figure 1B. According to these data, the Cu(2)HFAU zeolite contains Cu cations practically only in exchange positions (Cu^+^_exch._-CO band at 2158 cm^−1^), whereas, in Cu(5)NaFAU, zeolite comparable amounts of Cu^+^ in exchange (Cu^+^_exch._) and oxide forms (Cu^+^_ox._-CO band 2130 cm^−1^) were detected.

CO is an optimal probe molecule for Cu^+^, while NO is the most convenient probe for Cu^2+^. An elegant study of properties of Cu ions interacting with probe molecules (CO and NO) was realized by Palomino et al. [28]. The spectra of NO sorbed on our Cu(2)HFAU and Cu(5)NaFAU zeolites at 190 K are presented in Figure 1C. One hundred and ninety Kelvin was chosen as an optimal adsorption temperature, because at lower temperatures, NO interacts with the Lewis acid sites present in our zeolites, whereas, above 190K, NO oxidizes Cu^+^ to Cu^2+^. NO molecules sorbed at 190 K form Cu^2+^-NO (1885 cm^−1^) and Cu^+^_exch._(NO)_2_ dinitrosyls (1730 and 1825 cm^−1^) (Figure 1C). At higher temperatures, dinitrosyls decompose, forming mononitrosyls Cu^+^-NO (1815 cm^−1^ [27].

Direct comparison of the spectra presented in Figure 1C evidences that both Cu(2)HFAU and Cu(5)NaFAU contain Cu^2+^, the contribution of which is higher in Cu(5)NaFAU. The band of Cu^2+^-NO at 1885 cm^−1^ is rather broad, which suggests that it is composed of several maxima. According to Ziolek et al. [30], as well as of other authors [31,32], the exchange Cu^2+^ in square planar and square pyramidal coordination by framework oxygens are characterized by IR bands at 1880 and 1890 cm^−1^, whereas, for Cu^2+^ in CuO, the 1860 cm^−1^ band is typical [33]. Taking into account that our spectra were recorded at a low temperature (ca. 190 K) and, therefore, the NO IR bands are blue-shifted, it is possible that our broad band 1860–1910 cm^−1^ is the evidence of the presence of both exchange Cu^+^ and CuO. It seems possible that CuO was formed during the calcination of Cu(NO_3_)_2_, which was not consumed during the ion exchange.

**Figure 2:**
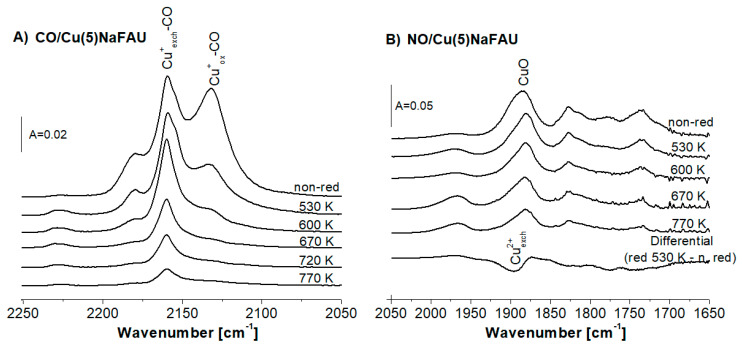
(**A**) The IR spectra of CO sorbed at room temperature on the Cu(5)NaFAU zeolite reduced by hydrogen at various temperatures. (**B**) The IR spectra of NO sorbed at 190 K on the Cu(5)NaFAU zeolite reduced by hydrogen at various temperatures. Bottom spectrum in Figure 3B is the difference between the spectrum of zeolite reduced at 530 K and the nonreduced sample.

Analysis of the results presented in Figure 1 and Figure 2 leads to the conclusion that zeolite Cu(5)NaFAU contains significantly smaller amounts of Cu accessible to probe molecules (most of Cu is inside the clusters). This zeolite contains important the contribution of Cu^+^ and Cu^2+^ in oxide forms.

**Figure 3:**
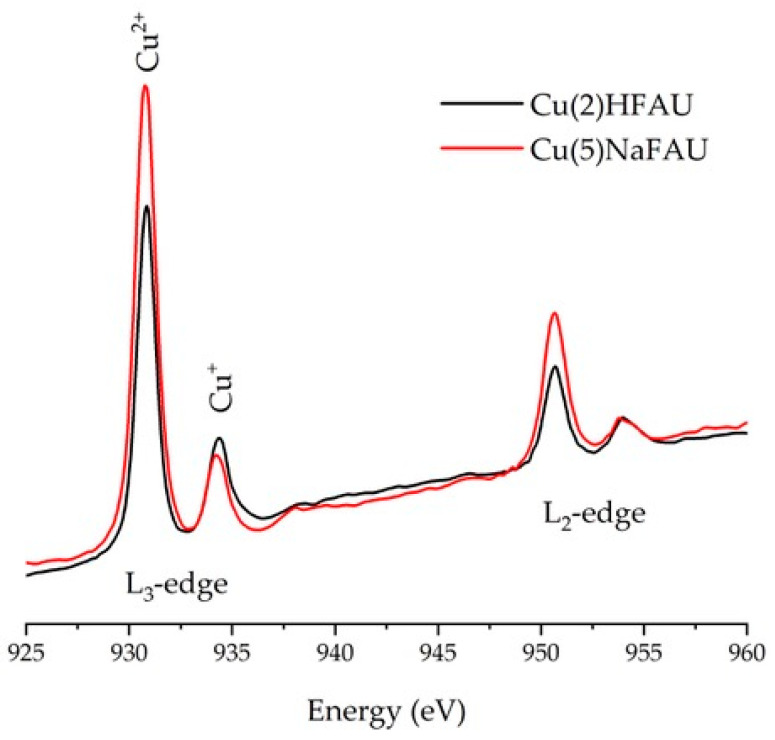
Partial fluorescence yield X-ray absorption spectroscopy (XAS) spectra of the Cu L-edge regions of Cu(2)HFAU and Cu(5)NaFAU zeolites.

Independently on IR studies, the status of Cu sites in our Cu zeolites was followed by X-ray absorption spectroscopy (XAS) at the Cu L-edge, which is an important tool for probing the properties of copper centers in transition-metal chemistry and catalysis [34,35,36]. In particular, L-edge X-ray absorption spectroscopy (XAS) probes transition from the metal 2s^2^p^6^ orbitals to 3D unoccupied electronic states that depend both on the oxidation state of the metal site and the local coordination environment [37]. It is proven that various copper ion oxidation states (Cu^+^ and Cu^2+^) can be identified and characterized based on XAS spectra [37,38,39,40]. The results obtained for Cu(2)HFAU and Cu(5)NaFAU are presented in Figure 3. By comparison with the literature, the lines at 930.8 eV and 950.6 eV should be associated with Cu^2+^ species, whereas those at 934.3 eV and 953.9 eV represent a lower oxidation state, such as Cu^+^. The data presented in Figure 3 indicate also that Cu(5)NaFAU contains more Cu^2+^ than Cu(2)HFAU (that agrees well with IR results obtained with NO as the probe molecules; Figure 1C); however, the quantitative analysis of the XAS results is difficult.

As mentioned in the Introduction, the process of reduction by hydrogen and ethanol, as well as the oxidation of Cu sites in Cu-faujasites, was followed by IR spectroscopy.

### 2.2. Reduction of Cu Species by Hydrogen

The process of the reduction of Cu species in our Cu(5)NaFAU zeolites by hydrogen was studied by IR spectroscopy in static conditions in situ in the IR cell. This experiment was performed in order to determine the relationship between the type of copper species and its susceptibility to the reduction with hydrogen. Additionally, the crystallinity of nonreduced and reduced Cu(5)FAU was investigated.

The reduction process of both the Cu^+^ and Cu^2+^ species (in exchange and oxide forms) by hydrogen was investigated for Cu(5)NaFAU, which was heated in hydrogen at 530, 600, 670, 720 and 770 K. Subsequently, the properties of the reduced Cu^+^ and Cu^2+^ were determined by the adsorption of CO or NO, respectively. Cu(5)NaFAU was chosen, because it contains comparable amounts of Cu^+^ in exchange and oxide forms, as well as notable amounts of Cu^2+^.

The IR spectra of CO sorbed at room temperature on Cu(5)NaFAU reduced at various temperatures are presented in Figure 2A. The Cu^+^_ox._-CO (2130 cm^−1^) band decreases in the first order before Cu^+^_exch._ (CO band 2158 cm^−1^). It evidences that Cu^+^_ox._ is more prone for the reduction than Cu^+^_exch._

The information on the reduction of the Cu^2+^ species was obtained from IR experiments of NO sorption at 190 K. The spectra are presented in Figure 2B. The intensity of the Cu^2+^-NO band at 1885 cm^−1^ decreased with the reduction temperature due to the reduction of Cu^2+^ species. Furthermore, we showed the difference of the spectrum recorded upon the reduction at 530 K minus the spectrum before treatment in Figure 2B (bottom line). Direct comparison between the spectra obtained for the samples reduced by hydrogen at various temperatures and the appearance of the differential spectrum (bottom line) led to the conclusion that the 1885 cm^−1^ band of Cu^2+^-NO is composed of two submaxima: 1880 and 1895 cm^−1^ assigned [27] to Cu^2+^_ox._ (CuO) and to Cu^2+^_exch._, respectively. The analysis of the IR spectra of NO sorbed (Figure 2B) led to the conclusion that the most prone to the reduction are Cu^2+^_exch._, whereas_._ CuO was found to be more resistant. The fact that Cu^2+^_exch._ is prone to the reduction agrees with the results obtained in our study on the reduction of Cu in CuY (Si/Al = 2,5) [41].

Summing up, the data presented in Figure 2A,B evidenced that the most prone to reduction are Cu^+^_ox._ and Cu^2+^_exch._ In turn, Cu^+^_exch._ and CuO seem to be more resistant for the reduction with hydrogen.

**Figure 4:**
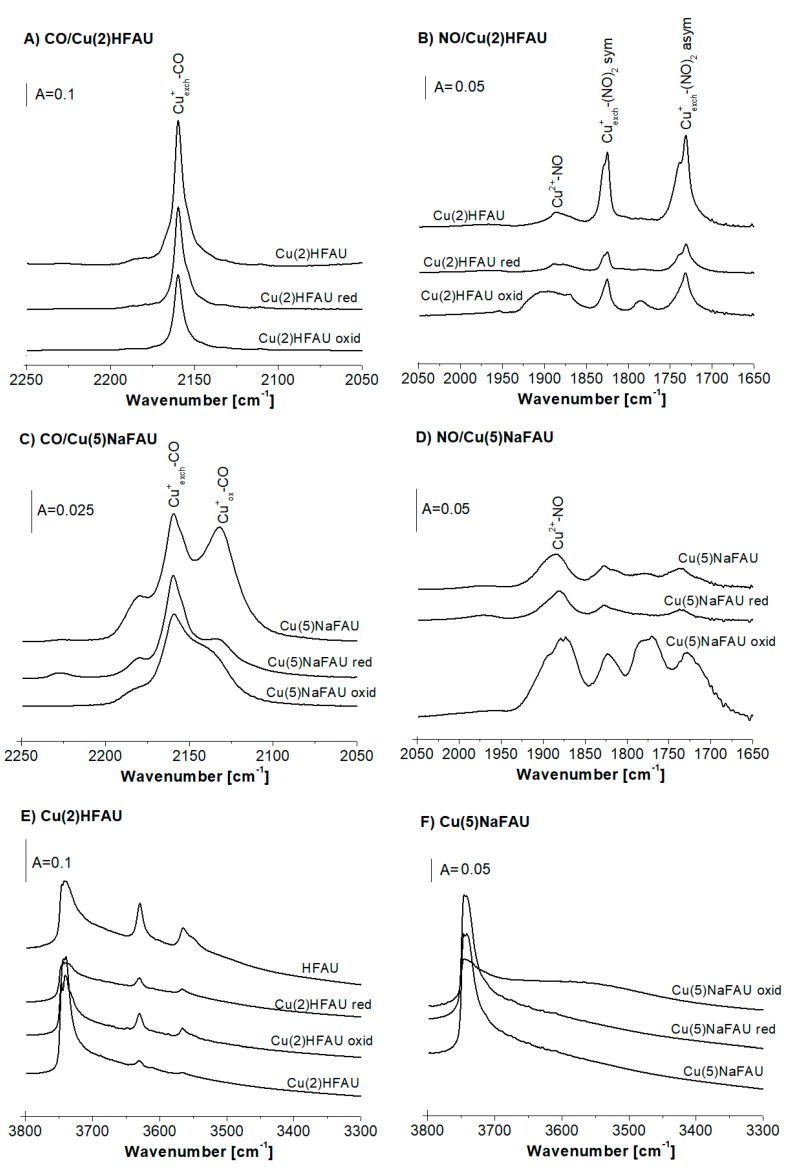
(**A**–**D**) The IR spectra of CO and NO sorbed at room temperature (CO) and at 190 K (NO) on Cu(2)HFAU and Cu(5)NaFAU zeolites, and the same samples reduced in hydrogen at 570 K and oxidized in oxygen at 570 K. (**E**,**F**) The IR spectra of the OH groups in Cu(2)HFAU (**E**) and in the Cu(5)NaFAU zeolite (**F**).

The data concerning the reduction of Cu species in Cu(2)HFAU and Cu(5)NaFAU by hydrogen at 570 K are presented in Figure 4. The results of CO and NO sorption on Cu(2)HFAU (Figure 4A,B) evidenced that the amount of Cu^+^_exch._ decreased by ca. 30% and the amount of Cu^2+^ decreased by 40–50% upon reduction. The data concerning the OH groups in this zeolite are given in Figure 4E. The introduction of Cu into the protonic form of faujasite (HFAU) caused a significant decrease the amount of acidic Si-OH-Al groups (IR bands 3550 and 3630 cm^−1^) due to the substitution of protons by Cu ions. On the other hand, the reduction by H_2_ resulted in an increase of the Si-OH-Al band according to the equation: 2Cu^+^ + H_2_ = 2Cu^0^ + 2H^+^.

The data on the reduction of Cu species in Cu(5)NaFAU are presented in Figure 4C,D. The treatment of this zeolite with hydrogen at 570 K causes an important decrease of the amount of Cu^+^_ox._ (IR band 2130 cm^−1^), a lowering of the Cu^+^_exch._ content (by ca. 20%) (Figure 4C) and a relatively small drop in the amount of Cu^2+^ (Figure 4D). As mentioned above, a high-frequency component of the Cu^2+^-NO band decreases, first of all suggesting that Cu^2+^_exch._ is more prone to the reduction by hydrogen than CuO. The treatment with hydrogen did not cause the change of the spectrum in the region of the Si-OH-Al groups (Figure 4F). This zeolite was obtained from sodium form NaFAU by the impregnation; therefore, it did not contain acidic hydroxyls. The reduction in hydrogen did not change the situation.

The XRD experiments evidenced that the reduction of Cu zeolites by hydrogen at 570 K did not deteriorate the zeolite crystallinity. Normally, the reduction of metal cations in zeolites by hydrogen produces metal clusters and protons. For CuY [20] and CuZSM-5 [29] zeolites, the reduction of Cu ions by hydrogen resulted in the formation of Cu^0^ (Cu^0^-CO IR bands 2124 cm^−1^ for CuZSM-5 and 2108 cm^−1^ for CuY). In our case, these bands were not observed (Figure 4). Two explanations can be considered. The first one assumes that Cu^0^ is situated inside cuboctahedra, being not accessible to probe molecules. The second explanation assumes that, under our experimental conditions, copper atoms form big agglomerates, in which only a small amount of Cu atoms is accessible to probe molecules.

### 2.3. Reduction of Cu Sites by Ethanol

**Figure 5:**
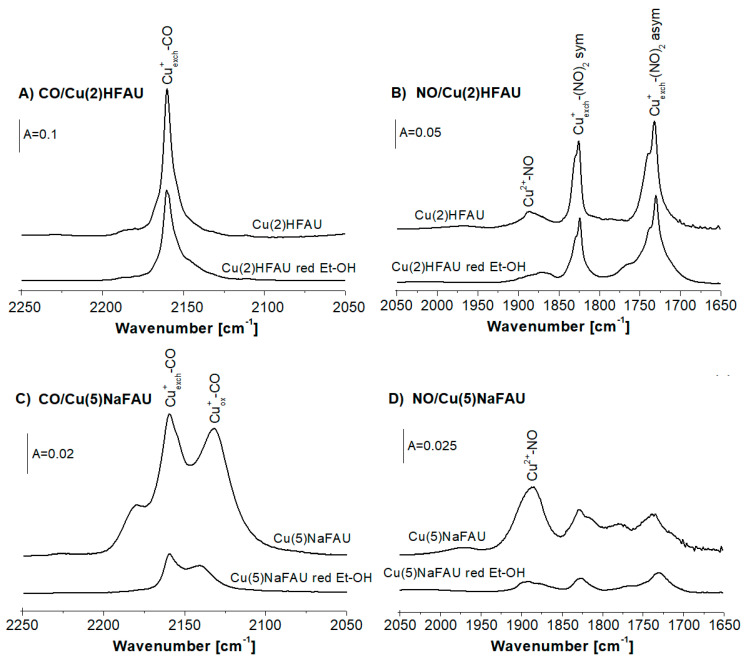
The IR spectra of CO sorbed at room temperature (**A**), NO sorbed at 190K (**B**) on the Cu2(H)FAU zeolite and the same sample reduced by ethanol. The IR spectra of CO sorbed at room temperature (**C**), NO sorbed at 190K (**D**) on the Cu(5)NaFAU zeolite and the same sample reduced by ethanol.

The process of the reduction of the Cu species by ethanol was followed using CO and NO as the probe molecules. The IR spectra of CO sorbed at room temperature and, of NO, sorbed at 190 K in nonreduced Cu(2)HFAU and Cu(5)FAU zeolites, as well as in those zeolites treated with ethanol vapors at 570 K, are presented in Figure 5. The reaction between Cu zeolites and ethanol causes the decrease of all the bands of Cu^+^-CO and Cu^2^-NO, evidencing the reduction of the Cu species. The most significant effect concerns Cu^2+^ in the oxide form (CuO) (Figure 5D). The transformation of ethanol in Cu zeolites will be the subject of our further studies.

### 2.4. Oxidation of Cu Sites in Zeolites by Oxygen

In the IR experiments, the Cu(2)HFAU and Cu(5)NaFAU zeolites were treated in situ in the IR cell with oxygen at 570 K. Subsequently, the cell was evacuated, and then, CO or NO were sorbed at room temperature (CO) or at 190K (NO). The spectra of the probe molecules sorbed on oxidized zeolites, as well as on the parent samples, are presented in Figure 4A–D. For the Cu(2)HFAU zeolite, the treatment with oxygen causes the decrease of the band of Cu^+^_exch._–CO (2158 cm^−1^) (Figure 4A) and an increase of the band of Cu^2+^–NO (ca. 1890 cm^−1^) (Figure 4B). These results indicate that Cu^+^_exch._ was oxidized to Cu^2+^. The band of Cu^2+^–NO in the oxidized zeolite is broader than in the parent sample (Figure 4B), which suggests that the broader spectrum of Cu^2+^ ions of various electro-acceptor properties are present in our oxidized sample.

The Cu(5)FAU zeolite contains much smaller amounts of Cu^+^_exch._ than Cu(2)FAU, and the amount of Cu^+^_ox._ is comparable with the Cu^+^_exch._ content. This zeolite contains also significant amounts of Cu^2+^. The treatment of this zeolite with oxygen at 570K causes some decrease of both the Cu^+^_exch._–CO (2158 cm^−1^) and Cu^+^_ox._–CO (ca. 2130 cm^−1^) bands and an increase of the band of Cu^2+^–NO (ca 1890 cm^−1^) (Figure 4C,D), which indicates the oxidation of the Cu^+^ sites to Cu^2+^. The maximum of Cu^2+^–NO, which shifts from 1885 cm^−1^ in the parent zeolite to 1875 cm^−1^ in the oxidized sample, indicates that the oxidation of the Cu^+^ species produces mostly CuO characterized by a NO frequency below 1880 cm^−1^.

The analysis of the spectra of the OH groups leads to the conclusion that the oxidation of the Cu^+^ species does not result in a visible variation of the amount of acidic Si-OH-Al groups.

### 2.5. Oxidation of Cu Sites in Zeolites by NO

**Figure 6:**
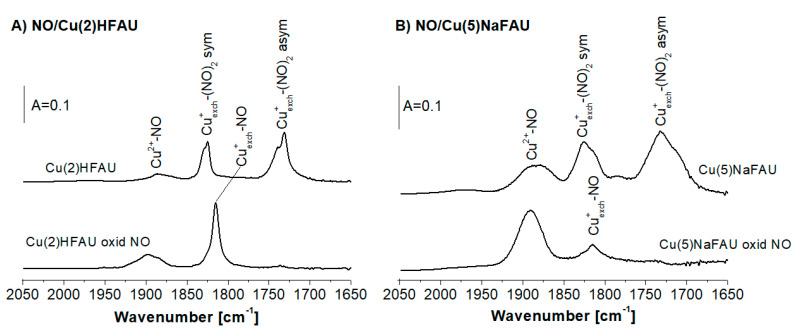
The IR spectra of NO sorbed at 190 K on the Cu(2)HFAU (**A**) and Cu(5)NaFAU zeolites (**B**). All IR spectra were recorded at 190 K and upon heating to room temperature (contact time was 27 h).

The results concerning the oxidation of the Cu^+^ species by NO are presented in Figure 6. For both Cu(2)HFAU and Cu(5)NaFAU, the sorption of NO at 190 K produced dinitrosyls (1730 and 1825 cm^−1^) and Cu^2+^-NO adducts (band at ca. 1880 cm^−1^). Dinitrosyls were transformed into mononitrosyls (NO band 1815 cm^−1^) if the temperature was raised to room temperature. The bands of the Cu^2+^-NO adducts increased after 27 h of contact between the zeolites and NO, indicating that NO acts as an oxidizer for the Cu^+^ species producing Cu^2+^.

## 3. Materials and Methods

### 3.1. Catalyst Preparation

Pristine zeolite with faujasite-type structure denoted as HFAU (Si/Al = 31) was supplied by Zeolyst International Company, Conshohocken, PA, USA (CBV 760). It was dealuminated by steaming and acid treatment by the producer. Cu-containing zeolites Cu(2)HFAU and Cu(5)NaFAU were obtained by the impregnation method with 0.5-M Cu(NO_3_)_2_ solution. Zeolite Cu(2)HFAU was obtained by the impregnation of pristine HFAU. It contained 2 wt.% of Cu. In order to obtain Cu(5)NaFAU, zeolite HFAU was first transformed into the sodium form by fivefold exchange with 0.5-M NaNO_3_ followed by washing in distilled water. NaFAU was subsequently impregnated with 0.5-M Cu(NO_3_)_2_, and zeolite containing 5 wt.% of Cu was obtained.

All samples were dried at 390 K and next calcined at 770 K.

### 3.2. IR Studies

IR studies were realized in transmission mode in in-house-fabricated vacuum IR cells. Prior to IR experiments, zeolites were evacuated in situ in the cell at 720 K for 1 h. The spectra were recorded with a NICOLET 6700 spectrometer (Thermo Scientific, Cambridge, MA, USA), with the spectral resolution of 1 cm^−1^. CO and NO (Air Products) were used as probe molecules. The adsorption of CO was performed at room temperature. The adsorption of NO was done at ca. 190 K.

The reduction by hydrogen or by ethanol was realized by the admission of H_2_ (ca. 300 Torr) or ethanol to the cell containing zeolite wafer pretreated in vacuum at 720 K. The zeolite was contacted with reducers at 570 K for 1 h. Next, the cell with zeolite was evacuated at 570 K for 1 h.

The oxidation by oxygen was realized by the admission of O_2_ (ca. 300 Torr) to the cell containing zeolite wafer pretreated in vacuum at 720 K. The zeolite was contacted with oxygen at 570 K for 1 h. Next, the cell with zeolite was evacuated at 570 K for 1 h.

The oxidation by NO was realized by the adsorption of NO at 170 K until the intensities of the dinitrosyl bands (1730 and 1825 cm^−1^) attained maximal intensities. The cell with adsorbed NO was subsequently heated to room temperature, and IR spectrum was recorded.

### 3.3. XAS Studies

Measurements of x-ray adsorption spectra (XAS) at the Cu L_2_ and L_3_ edges were performed at the National Synchrotron Radiation Centre SOLARIS in Krakow at the bending magnet XAS/PEEM beamline [42]. The spectra were collected at the XAS end station in the partial fluorescent yield (PFY) detection mode using a silicon drift detector. The x-ray energy in the Cu L-edges range was calibrated with an accuracy of ±0.3 eV.

### 3.4. XRD Studies

The powder X-ray diffraction (XRD) measurements were carried out using a PANalytical Cubix X’Pert Pro diffractometer, with CuK_α_ radiation, λ = 1.5418 Å in the 2θ angle range of 2–40°. Both Cu(2)HFAU and Cu(5)NaFAU zeolites were pretreated in vacuum at 720K for 1 h, and hydrogen was next admitted to the cell at 570 K for 1 h. The diffractograms of zeolites were reduced, and zeolites vacuum-treated but not reduced were compared.

## 4. Conclusions

Four kinds of Cu species (Cu^+^_exch.,_ Cu^+^_ox_, Cu^2+^_exch._ and Cu^2+^_ox_ (CuO)) were found in the CuFAU zeolites, in which Cu was introduced by the impregnation of dealuminated zeolites of faujasite types (Si/Al = 31). The copper contents in Cu(2)HFAU and Cu(5)NaFAU were 2 wt.% and 5 wt.%, respectively. Cu(2)HFAU contained mostly Cu^+^_exch._, whereas, in Cu(5)NaFAU, mostly the oxide forms Cu^+^_ox._ and Cu^2+^_ox._ were found. The processes of reduction (by hydrogen or ethanol) and oxidation (by oxygen or NO) of the Cu^+^ and Cu^2+^ species in dealuminated faujasite were followed by IR spectroscopy with CO and NO as the probe molecules. CO sorption experiments evidenced that Cu^+^_ox._ was more prone to reduction by hydrogen than Cu^+^_exch._ On the other hand, NO sorption studies proved that Cu^2+^_exch._ was more susceptible to reduction than Cu^2+^_ox._ (CuO). The treatment with ethanol at 570 K reduced mostly the Cu^2+^ species. The treatment with oxygen at 570 K, as well as the interaction with NO at room temperature, led to the production of Cu^2+^ at the expense of Cu^+^.
